# Supplementary material for: The Association of Dietary Polyamines with Mortality and the Risk of Cardiovascular Disease: A Prospective Study in UK Biobank
Source: Nutrients. 2024 Dec 16;16(24):4335. doi: 10.3390/nu16244335 (PMC11678356; doi:10.3390/nu16244335)
Supplement: Supplementary file 1 [file nutrients-16-04335-s001.zip › Supplementary Material-Figure.pdf]

Supplementary Material

CONTENTS

Figure S1. Flow chart of the study population-- 2

Figure S2. Association between spermidine intake and all-cause mortality or incident CVD-----3

Figure S3. Association between spermine intake and all-cause mortality or incident CVD-------4

Figure S4. Association between putrescine intake and all-cause mortality or incident CVD-----5

Figure S5. Association between dietary spermidine intake and all-cause mortality or incident CVD and components of CVD (CVD death, CHD and stroke) ---6,7

Figure S6. Association between dietary spermine intake and all-cause mortality or incident CVD and components of CVD (CVD death, CHD and stroke) ---8,9

Figure S7. Association between dietary putrescine intake and all-cause mortality or incident CVD and components of CVD (CVD death, CHD and stroke) --10,11

Figure S8. Association between dietary spermidine intake and CVD death stratified by potential risk factors ---12

Figure S9. Association between dietary spermine intake and CVD death stratified by potential risk factors ---13

Figure S10. Association between dietary putrescine intake and CVD death stratified by potential risk factors ---14

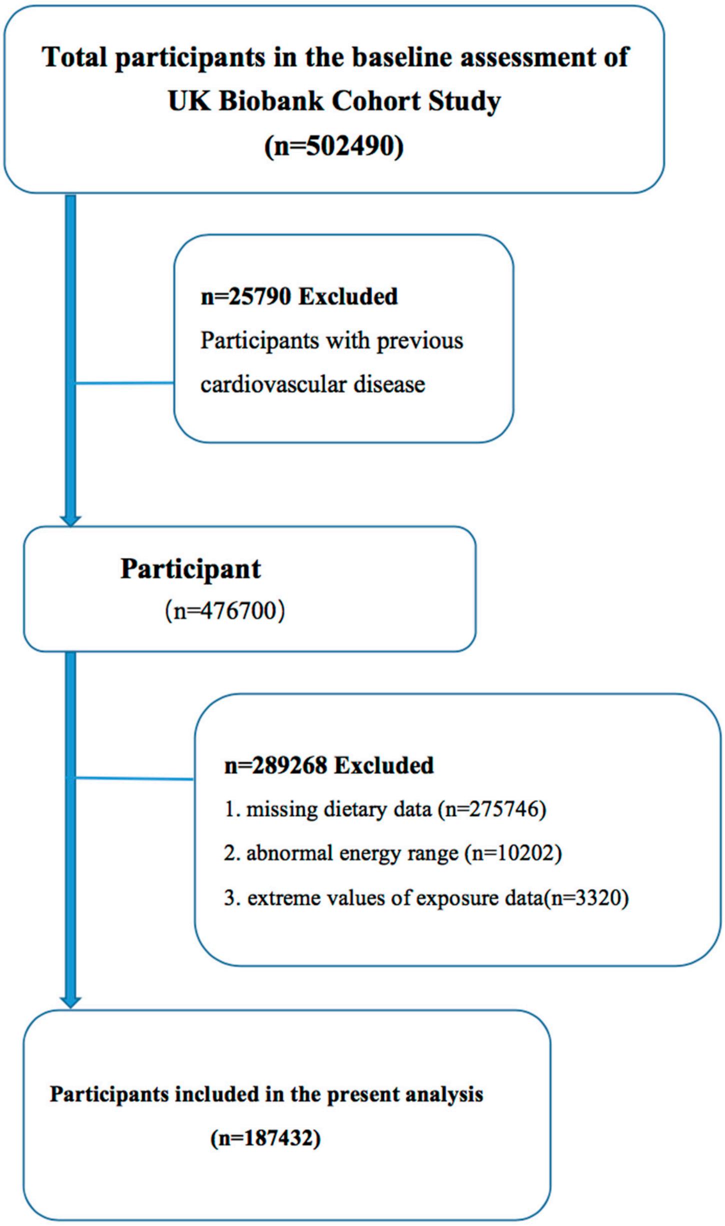

Figure S1: Flowchart of participant selection.

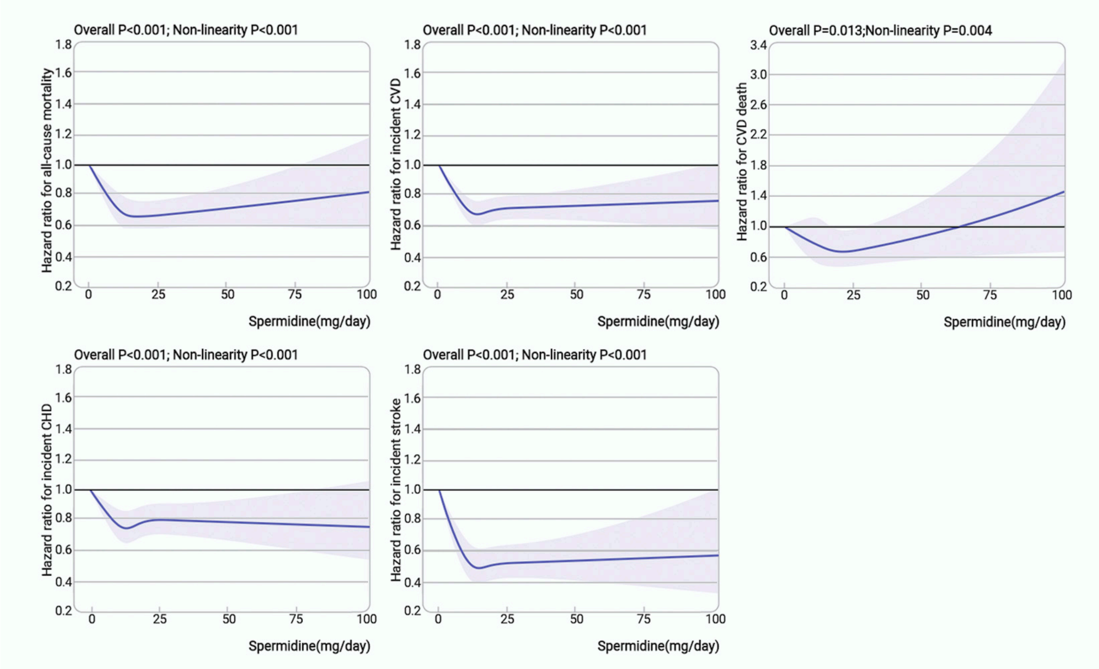

Figure S2 Association between dietary spermidine intake and all-cause mortality or incident CVD. Knots were placed at the 25th, 50th, 75th, and 100th percentiles of the spermidine intake distribution. Analyses adjusted for age, sex, ethnicity, Townsend deprivation index, education level, systolic blood pressure, body mass index, physical activity, smoking status, alcohol status, sleep duration, energy, hypertension, diabetes, hypercholesteremia, antihypertensive treatment, lipid treatment, insulin treatment. Components of CVD (CVD death, CHD and stroke) were also analyzed. Shaded areas represent 95% confidence intervals.

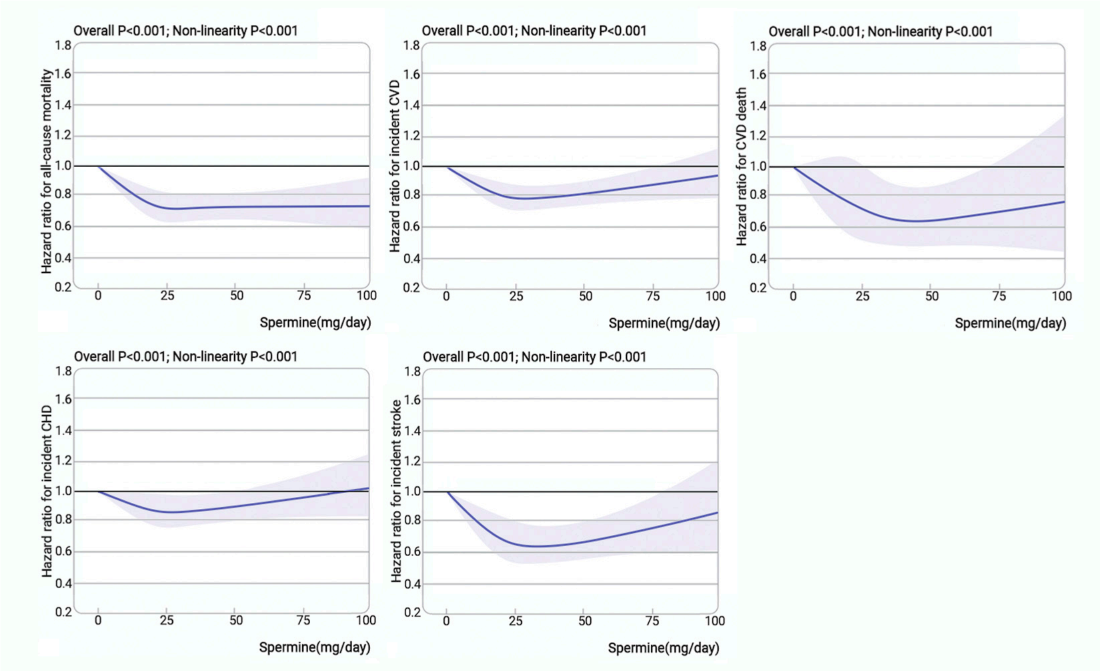

Figure S3 Association between dietary spermine intake and all-cause mortality or incident CVD. Knots were placed at the 25th, 50th, 75th, and 100th percentiles of the spermine intake distribution. Analyses adjusted for age, sex, ethnicity, Townsend deprivation index, education level, systolic blood pressure, body mass index, physical activity, smoking status, alcohol status, sleep duration, energy, hypertension, diabetes, hypercholesteremia, antihypertensive treatment, lipid treatment, insulin treatment. Components of CVD (CVD death, CHD and stroke) were also analyzed. Shaded areas represent 95% confidence intervals.

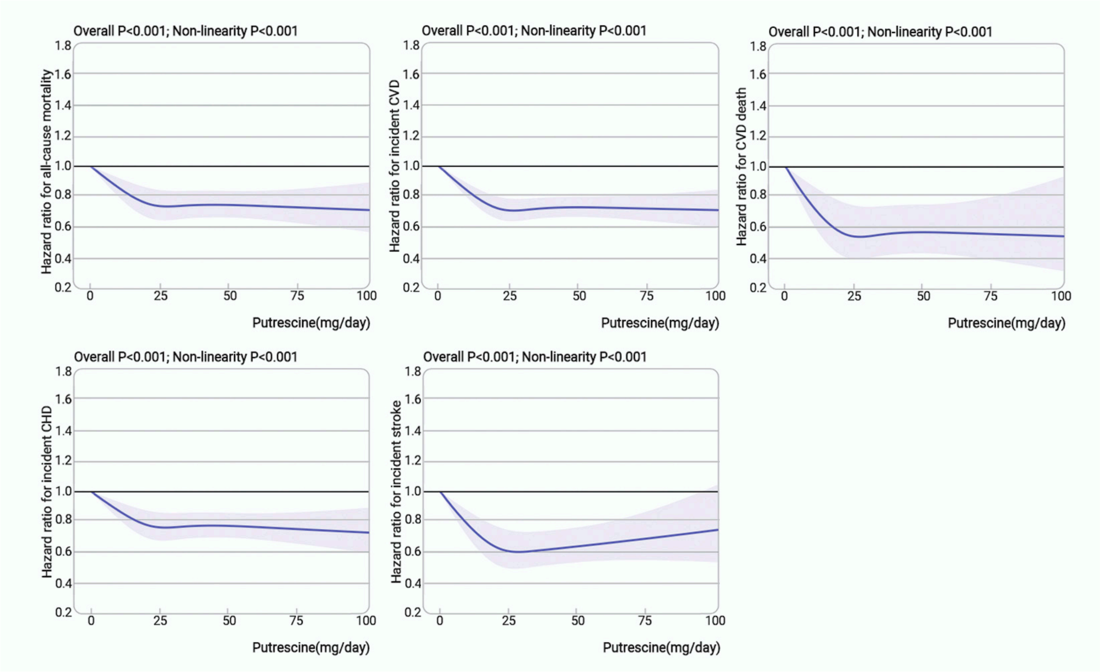

Figure S4 Association between dietary putrescine intake and all-cause mortality or incident CVD. Knots were placed at the 25th, 50th, 75th, and 100th percentiles of the putrescine intake distribution. Analyses adjusted for age, sex, ethnicity, Townsend deprivation index, education level, systolic blood pressure, body mass index, physical activity, smoking status, alcohol status, sleep duration, energy, hypertension, diabetes, hypercholesteremia, antihypertensive treatment, lipid treatment, insulin treatment. Components of CVD (CVD death, CHD and stroke) were also analyzed. Shaded areas represent 95% confidence intervals.

Outcomes		No.of events	Person-years	Incidence rate per 1000 person-years	Model 1 Hazard ratio(95%CI)	Model 2 Hazard ratio(95%CI)
**All-cause mortality**						
Spermidine	Q1	1555	428473	3.63	Ref.	Ref.
	Q2	1346	431564	3.12	0.79(0.74-0.85)	0.84(0.78-0.90)
	Q3	1424	431590	3.3	0.79(0.74-0.85)	0.87(0.81-0.94)
	Q4	1454	431109	3.37	0.78(0.73-0.84)	0.86(0.80-0.93)
	Q5	1569	430755	3.65	0.81(0.75-0.87)	0.83(0.77-0.89)
**Incident CVD**						
Spermidine	Q1	2491	418386	5.95	Ref.	Ref.
	Q2	2281	423355	5.39	0.85(0.80-0.90)	0.9(0.85-0.95)
	Q3	2366	423083	5.59	0.84(0.79-0.89)	0.9(0.85-0.96)
	Q4	2420	422622	5.73	0.83(0.79-0.88)	0.9(0.85-0.95)
	Q5	2758	420145	6.56	0.91(0.86-0.96)	0.94(0.89-1.00)
**CVD death**						
Spermidine	Q1	250	428473	0.58	Ref.	Ref.
	Q2	224	431564	0.52	0.82(0.68-0.98)	0.87(0.73-1.04)
	Q3	215	432032	0.5	0.74(0.61-0.88)	0.8(0.66-0.97)
	Q4	224	431492	0.52	0.74(0.62-0.89)	0.8(0.67-0.97)
	Q5	263	430410	0.61	0.82(0.69-0.98)	0.84(0.70-1.01)
**CHD**						
Spermidine	Q1	1800	420189	4.28	Ref.	Ref.
	Q2	1690	424704	3.98	0.87(0.82-0.94)	0.81(0.72-0.91)
	Q3	1785	424520	4.2	0.88(0.83-0.94)	0.76(0.68-0.85)
	Q4	1809	424152	4.26	0.87(0.81-0.93)	0.78(0.69-0.88)
	Q5	2052	421904	4.86	0.95(0.89-1.01)	0.84(0.75-0.95)
**Stroke**						
Spermidine	Q1	642	425877	1.51	Ref.	Ref.
	Q2	550	430062	1.28	0.78(0.70-0.88)	0.90(0.85-0.95)
	Q3	544	430392	1.26	0.73(0.65-0.82)	0.90(0.85-0.96)
	Q4	584	429741	1.36	0.75(0.67-0.84)	0.90(0.85-0.95)
	Q5	687	428390	1.6	0.85(0.76-0.95)	0.94(0.89-1.00)

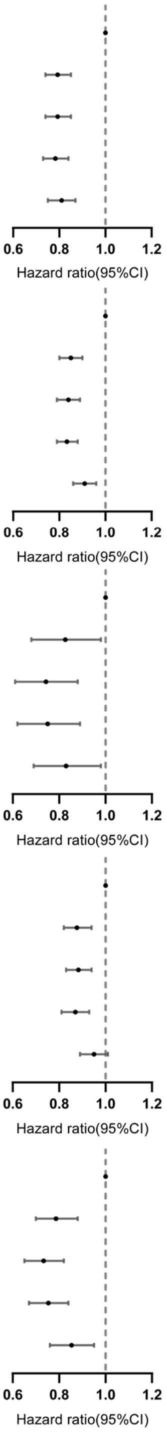

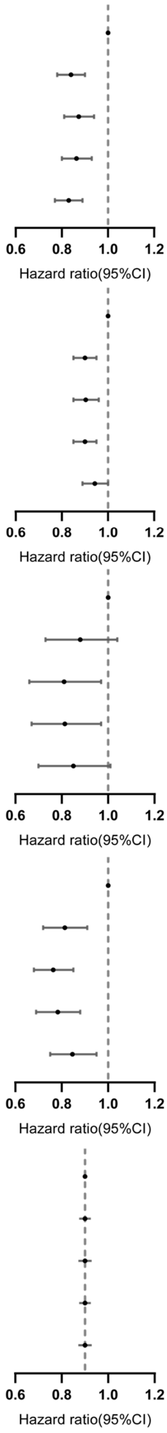

Figure S5 Association between dietary spermidine intake and all-cause mortality or incident CVD and components of CVD (CVD death, CHD and stroke).

Mode1 1: Analyses adjusted for age, sex.

Model 2: Analyses adjusted for age, sex, ethnicity, Townsend deprivation index, education level, systolic blood pressure, body mass index, physical activity, smoking status, alcohol status, sleep duration, energy, hypertension, diabetes, hypercholesteremia, antihypertensive treatment, lipid treatment, insulin treatment.

Outcomes			No.of events	Person-years	Incidence rate per 1000 person-years	Model 1 Hazard ratio(95%CI)	Model 2 Hazard ratio(95%CI)
**All-cause mortality**							
	Spermine	Q1	1515	429033	3.53	Ref.	Ref.
		Q2	1434	431298	3.32	0.89(0.83-0.96)	0.92(0.86-0.99)
		Q3	1412	431368	3.27	0.86(0.80-0.92)	0.89(0.82-0.95)
		Q4	1457	431191	3.38	0.87(0.81-0.93)	0.89(0.83-0.96)
		Q5	1539	430601	3.55	0.89(0.83-0.96)	0.90(0.84-0.98)
**Incident CVD**							
	Spermine	Q1	2433	419815	5.8	Ref.	Ref.
		Q2	2382	422545	5.64	0.92(0.87-0.97)	0.94(0.89-0.99)
		Q3	2328	422797	5.51	0.87(0.82-0.92)	0.89(0.84-0.95)
		Q4	2458	422173	5.82	0.89(0.85-0.95)	0.91(0.86-0.97)
		Q5	2715	420261	6.46	0.96(0.91-1.01)	0.96(0.91-1.02)
**CVD death**							
	Spermine	Q1	242	429033	0.56	Ref.	Ref.
		Q2	237	431298	0.55	0.91(0.76-1.084)	0.93(0.78-1.12)
		Q3	236	431368	0.55	0.87(0.73-1.04)	0.89(0.74-1.07)
		Q4	227	431191	0.53	0.81(0.68-0.97)	0.81(0.67-0.98)
		Q5	234	430601	0.54	0.80(0.67-0.96)	0.78(0.64-0.94)
**CHD**							
	Spermine	Q1	1750	421596	4.15	Ref.	Ref.
		Q2	1766	423977	4.17	0.95(0.89-1.01)	0.98(0.92-1.05)
		Q3	1731	424306	4.08	0.90(0.84-0.96)	0.93(0.87-0.99)
		Q4	1832	423630	4.32	0.93(0.87-0.99)	0.95(0.89-1.02)
		Q5	2057	421960	4.87	1.00(0.94-1.07)	1.01(0.95-1.09)
**Stroke**							
	Spermine	Q1	637	426999	1.49	Ref.	Ref.
		Q2	578	429642	1.35	0.85(0.76-0.95)	0.86(0.77-0.97)
		Q3	565	429663	1.31	0.81(0.72-0.91)	0.82(0.73-0.92)
		Q4	583	429555	1.36	0.82(0.73-0.92)	0.82(0.73-0.92)
		Q5	644	28603	1.5	0.88(0.79-0.99)	0.87(0.77-0.98)

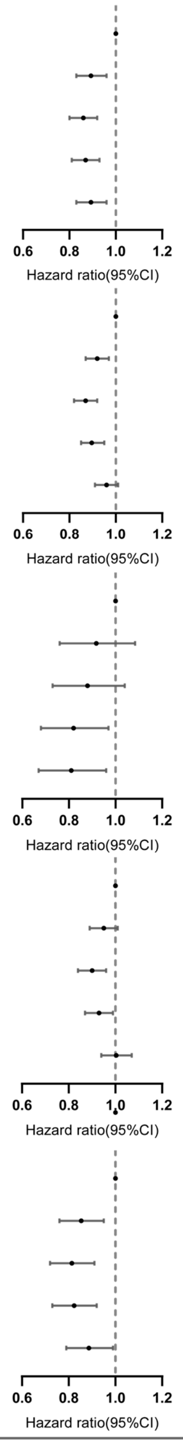

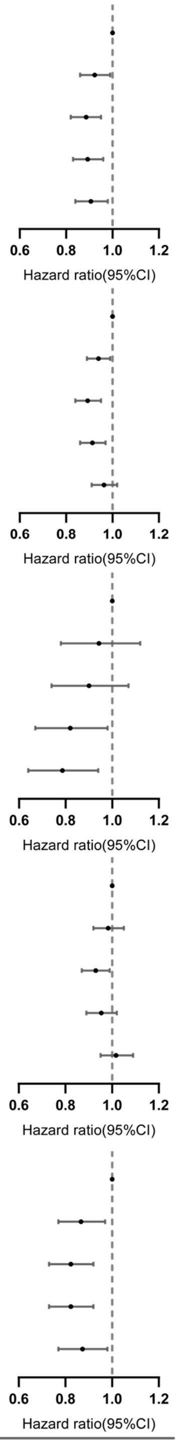

Figure S6 Association between dietary spermine intake and all-cause mortality or incident CVD and components of CVD (CVD death, CHD and stroke).

Mode1 1: Analyses adjusted for age, sex.

Model 2: Analyses adjusted for age, sex, ethnicity, Townsend deprivation index, education level, systolic blood pressure, body mass index, physical activity, smoking status, alcohol status, sleep duration, energy, hypertension, diabetes, hypercholesteremia, antihypertensive treatment, lipid treatment, insulin treatment.

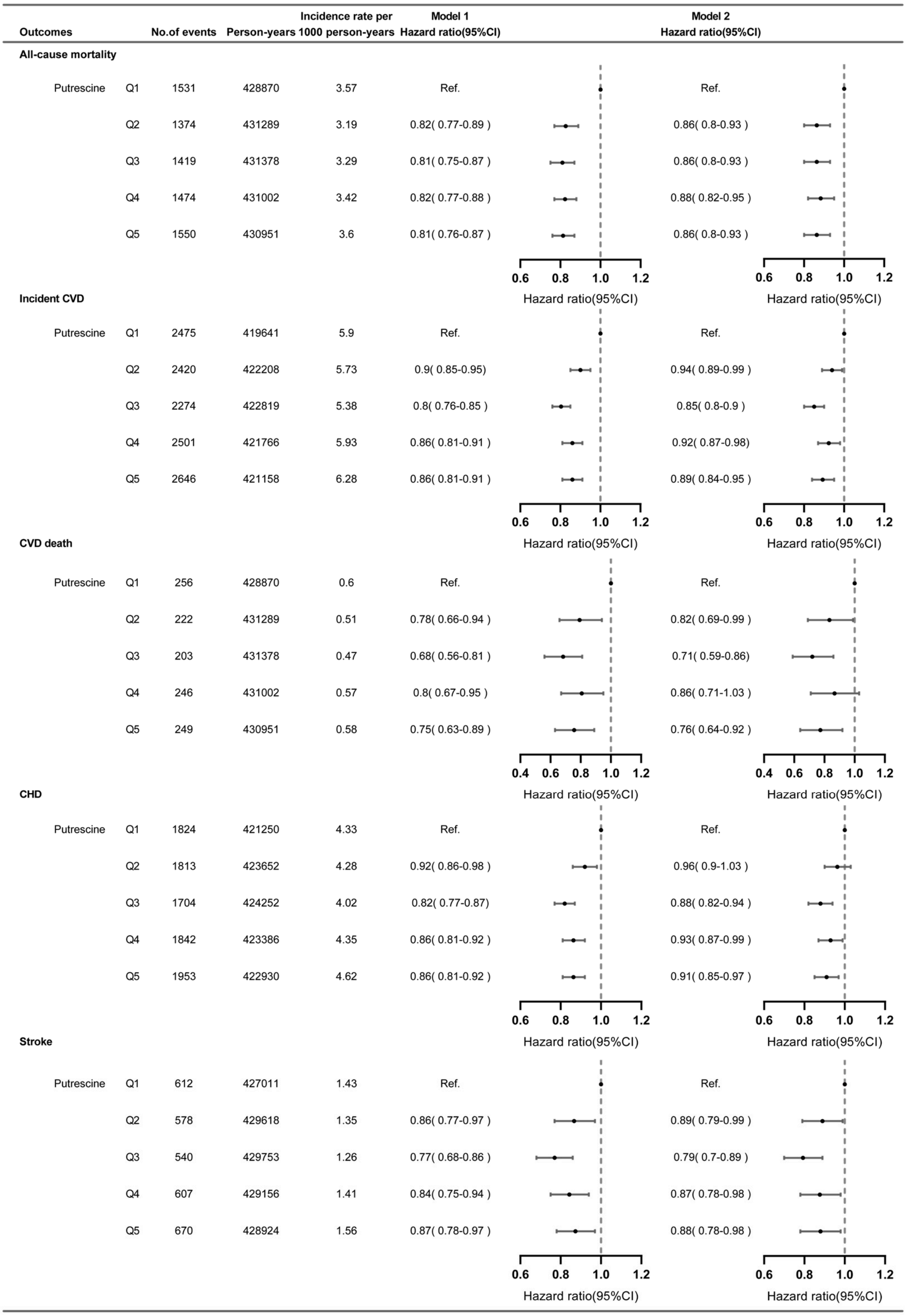

Outcomes			No.of events	Person-years	Incidence rate per 1000 person-years	Model 1 Hazard ratio(95%CI)	Model 2 Hazard ratio(95%CI)
**All-cause mortality**							
	Putrescine	Q1	1531	428870	3.57	Ref.	Ref.
		Q2	1374	431289	3.19	0.82(0.77-0.89)	0.86(0.8-0.93)
		Q3	1419	431378	3.29	0.81(0.75-0.87)	0.86(0.8-0.93)
		Q4	1474	431002	3.42	0.82(0.77-0.88)	0.88(0.82-0.95)
		Q5	1550	430951	3.6	0.81(0.76-0.87)	0.86(0.8-0.93)
**Incident CVD**							
	Putrescine	Q1	2475	419641	5.9	Ref.	Ref.
		Q2	2420	422208	5.73	0.9(0.85-0.95)	0.94(0.89-0.99)
		Q3	2274	422819	5.38	0.8(0.76-0.85)	0.85(0.8-0.9)
		Q4	2501	421766	5.93	0.86(0.81-0.91)	0.92(0.87-0.98)
		Q5	2646	421158	6.28	0.86(0.81-0.91)	0.89(0.84-0.95)
**CVD death**							
	Putrescine	Q1	256	428870	0.6	Ref.	Ref.
		Q2	222	431289	0.51	0.78(0.66-0.94)	0.82(0.69-0.99)
		Q3	203	431378	0.47	0.68(0.56-0.81)	0.71(0.59-0.86)
		Q4	246	431002	0.57	0.8(0.67-0.95)	0.86(0.71-1.03)
		Q5	249	430951	0.58	0.75(0.63-0.89)	0.76(0.64-0.92)
**CHD**							
	Putrescine	Q1	1824	421250	4.33	Ref.	Ref.
		Q2	1813	423652	4.28	0.92(0.86-0.98)	0.96(0.9-1.03)
		Q3	1704	424252	4.02	0.82(0.77-0.87)	0.88(0.82-0.94)
		Q4	1842	423386	4.35	0.86(0.81-0.92)	0.93(0.87-0.99)
		Q5	1953	422930	4.62	0.86(0.81-0.92)	0.91(0.85-0.97)
**Stroke**							
	Putrescine	Q1	612	427011	1.43	Ref.	Ref.
		Q2	578	429618	1.35	0.86(0.77-0.97)	0.89(0.79-0.99)
		Q3	540	429753	1.26	0.77(0.68-0.86)	0.79(0.7-0.89)
		Q4	607	429156	1.41	0.84(0.75-0.94)	0.87(0.78-0.98)
		Q5	670	428924	1.56	0.87(0.78-0.97)	0.88(0.78-0.98)

Figure S7 Association between dietary putrescine intake and all-cause mortality or incident CVD and components of CVD (CVD death, CHD and stroke).

Mode1 1: Analyses adjusted for age, sex.

Model 2: Analyses adjusted for age, sex, ethnicity, Townsend deprivation index, education level, systolic blood pressure, body mass index, physical activity, smoking status, alcohol status, sleep duration, energy, hypertension, diabetes, hypercholesteremia, antihypertensive treatment, lipid treatment, insulin treatment.

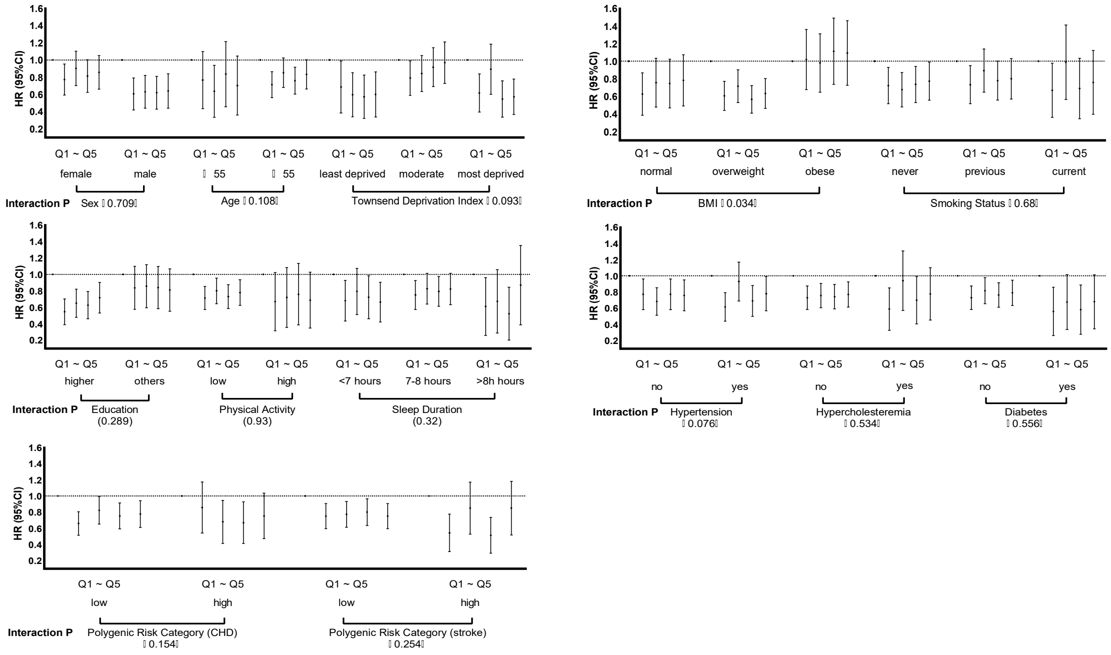

Figure S8 Association between dietary spermidine intake and CVD death stratified by potential risk factors. Analyses adjusted for age, sex, ethnicity, Townsend deprivation index, education level, systolic blood pressure, body mass index, physical activity, smoking status, alcohol status, sleep duration, energy, hypertension, diabetes, hypercholesteremia, antihypertensive treatment, lipid treatment, insulin treatment.

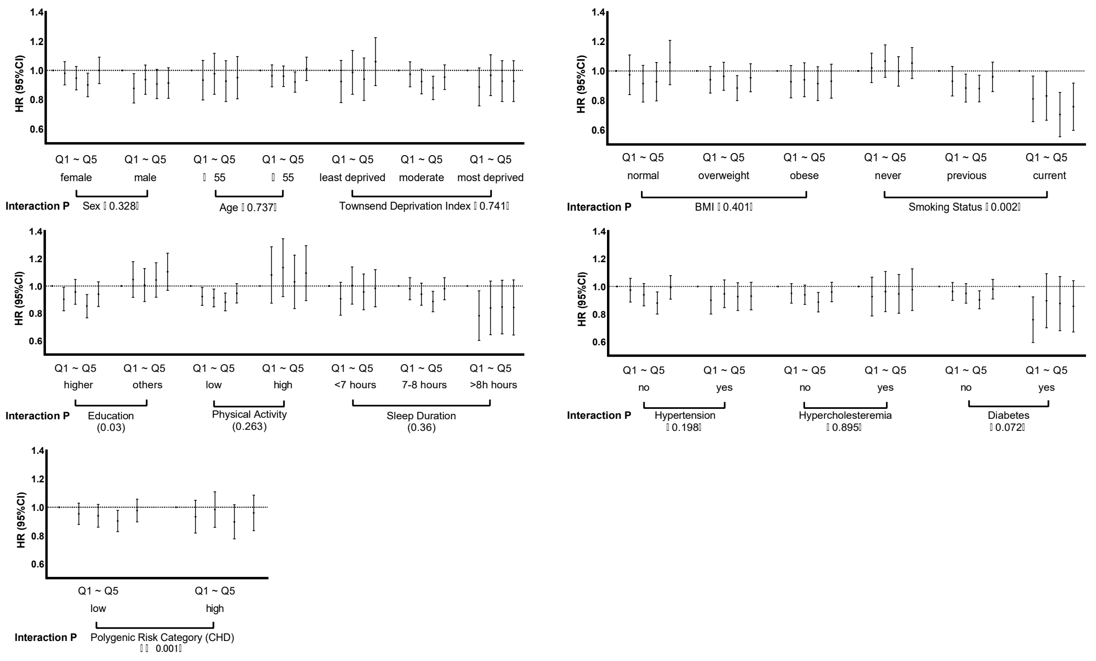

Figure S9 Association between dietary spermine intake and incident CHD stratified by potential risk factors. Analyses adjusted for age, sex, ethnicity, Townsend deprivation index, education level, systolic blood pressure, body mass index, physical activity, smoking status, alcohol status, sleep duration, energy, hypertension, diabetes, hypercholesteremia, antihypertensive treatment, lipid treatment, insulin treatment.

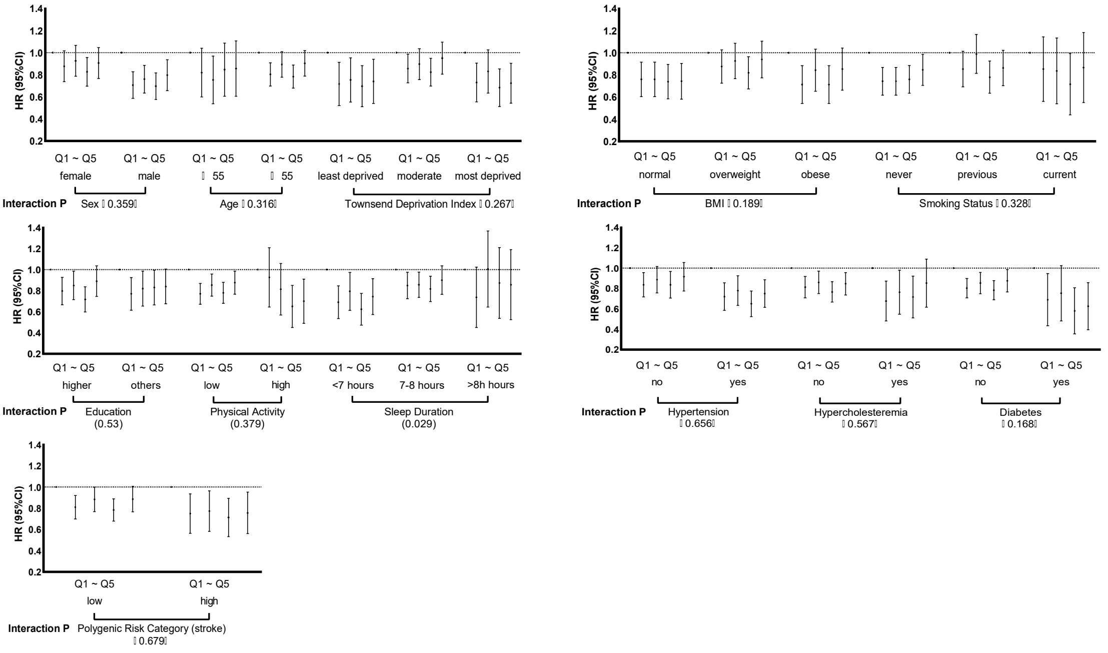

Figure S10 Association between dietary putrescine intake and incident stroke stratified by potential risk factors. Analyses adjusted for age, sex, ethnicity, Townsend deprivation index, education level, systolic blood pressure, body mass index, physical activity, smoking status, alcohol status, sleep duration, energy, hypertension, diabetes, hypercholesteremia, antihypertensive treatment, lipid treatment, insulin treatment.
