# Supplementary material for: The Association of Dietary Polyamines with Mortality and the Risk of Cardiovascular Disease: A Prospective Study in UK Biobank
Source: Nutrients. 2024 Dec 16;16(24):4335. doi: 10.3390/nu16244335 (PMC11678356; doi:10.3390/nu16244335)
Supplement: Supplementary file 1 [file nutrients-16-04335-s001.zip › Supplementary Material-Table.pdf]

Supplementary Material

CONTENTS

Table S1: sensitivity analysis: Excluding participants who developed endpoint within two years of follow-up------------ 2,3

Table S2: sensitivity analysis: Excluding participants with the top 5% and bottom 5% of polyamines----------------------- 4,5

Table S3: sensitivity analysis: adjusting for potential effect mediators (CHD and stroke predisposition score) ------------6,7

Table S4: sensitivity analysis: Excluding participants with any missing values of covariates ------------------------------------8,9

Sensitivity analysis Table S1: Excluding participants who developed endpoint within two years of follow-up.

		all-cause mortality		CVD events		CVD death		CHD		Stroke	
		**HR (95% CI)**	***P value***	**HR (95% CI)**	***P value***	**HR (95% CI)**	***P value***	**HR (95% CI)**	***P value***	**HR (95% CI)**	***P value***
**Dietary polyamines**	Quintile1	1.00 (ref.)	-	1.00 (ref.)	-	1.00 (ref.)	-	1.00 (ref.)	-	1.00 (ref.)	-
	Quintile2	0.82(0.76-0.88)	<0.001	0.89(0.84-0.95)	<0.001	0.68(0.56-0.83)	<0.001	0.94(0.88-1.01)	0.102	0.78(0.70-0.88)	<0.001
	Quintile3	0.87(0.81-0.94)	<0.001	0.90(0.85-0.96)	0.001	0.77(0.64-0.93)	0.006	0.95(0.89-1.02)	0.133	0.84(0.75-0.94)	0.003
	Quintile4	0.86(0.80-0.93)	<0.001	0.87(0.82-0.93)	<0.001	0.72(0.60-0.87)	0.001	0.91(0.85-0.97)	0.007	0.75(0.67-0.85)	<0.001
	Quintile5	0.83(0.77-0.89)	<0.001	0.92(0.87-0.98)	0.008	0.76(0.63-0.91)	0.003	0.97(0.90-1.04)	0.373	0.85(0.75-0.95)	0.006
**Spermidine**	Quintile1	1.00 (ref.)	-	1.00 (ref.)	-	1.00 (ref.)	-	1.00 (ref.)	-	1.00 (ref.)	-
	Quintile2	0.84(0.78-0.91)	<0.001	0.91(0.86-0.96)	0.001	0.87(0.72-1.04)	0.133	0.95(0.89-1.02)	0.157	0.82(0.73-0.92)	0.001
	Quintile3	0.86(0.80-0.93)	<0.001	0.91(0.86-0.97)	0.002	0.81(0.67-0.97)	0.025	0.97(0.91-1.04)	0.444	0.77(0.68-0.86)	<0.001
	Quintile4	0.85(0.79-0.92)	<0.001	0.91(0.86-0.97)	0.003	0.79(0.66-0.96)	0.015	0.97(0.91-1.04)	0.418	0.79(0.70-0.89)	<0.001
	Quintile5	0.85(0.78-0.91)	<0.001	0.95(0.90-1.01)	0.105	0.84(0.70-1.01)	0.069	1.01(0.94-1.08)	0.842	0.85(0.76-0.96)	0.008
**Spermine**	Quintile1	1.00 (ref.)	-	1.00 (ref.)	-	1.00 (ref.)	-	1.00 (ref.)	-	1.00 (ref.)	-
	Quintile2	0.93(0.86-1.00)	0.05	0.95(0.90-1.01)	0.079	0.93(0.77-1.11)	0.413	0.99(0.92-1.06)	0.709	0.86(0.77-0.97)	0.013
	Quintile3	0.9(0.83-0.97)	0.004	0.90(0.85-0.96)	0.001	0.89(0.74-1.07)	0.219	0.94(0.88-1.01)	0.094	0.82(0.73-0.92)	0.001
	Quintile4	0.9(0.83-0.97)	0.005	0.92(0.87-0.98)	0.007	0.82(0.68-0.99)	0.042	0.96(0.90-1.03)	0.263	0.83(0.74-0.93)	0.002

	Quintile5	0.91(0.84-0.98)	0.014	0.97(0.91-1.02)	0.247	0.77(0.64-0.94)	0.011	1.02(0.95-1.09)	0.584	0.88(0.78-0.99)	0.027
**Putrescine**	Quintile1	1.00 (ref.)	-	1.00 (ref.)	-	1.00 (ref.)	-	1.00 (ref.)	-	1.00 (ref.)	-
	Quintile2	0.87(0.81-0.94)	<0.001	0.95(0.89-1.00)	0.061	0.84(0.70-1.00)	0.054	0.97(0.90-1.03)	0.332	0.90(0.80-1.01)	0.078
	Quintile3	0.87(0.81-0.94)	<0.001	0.86(0.81-0.91)	<0.001	0.73(0.61-0.89)	0.001	0.88(0.82-0.94)	<0.001	0.80(0.71-0.90)	<0.001
	Quintile4	0.89(0.82-0.96)	0.002	0.93(0.88-0.99)	0.019	0.87(0.72-1.04)	0.129	0.94(0.88-1.01)	0.085	0.88(0.79-0.90)	0.04
	Quintile5	0.86(0.80-0.93)	<0.001	0.90(0.85-0.96)	0.001	0.77(0.64-0.93)	0.005	0.92(0.86-0.98)	0.015	0.88(0.79-0.99)	0.039

Cox proportional hazards regression adjusted for age, sex, Ethnicity (White European, mixed, Asian, black, others), Townsend deprivation index (least deprived, moderate deprived, most deprived), Education status (College or university degree, others), Systolic Blood Pressure, Body mass index, Physical activity (low and high level), Smoking status (Never, Former, Current), alcohol status (Never, Former, Current), sleep duration(<7h, 7-8h, >8h), energy, Hypertension, Diabetes, Hypercholesteremia, Drug use (Antihypertensive, Lipid treatment, Insulin treatment)

Sensitivity analysis Table S2: Excluding participants with the top 5% and bottom 5% of polyamines.

		all-cause mortality		CVD events		CVD death		CHD		Stroke	
		**HR (95% CI)**	***P value***	**HR (95% CI)**	***P value***	**HR (95% CI)**	***P value***	**HR (95% CI)**	***P value***	**HR (95% CI)**	***P value***
**Dietary polyamines**	Quintile1	1.00 (ref.)	-	1.00 (ref.)	-	1.00 (ref.)	-	1.00 (ref.)	-	1.00 (ref.)	-
	Quintile2	0.83(0.77-0.9)	<0.001	0.94(0.88-0.99)	0.045	0.68(0.55-0.82)	<0.001	0.99(0.92-1.07)	0.79	0.84(0.74-0.95)	0.007
	Quintile3	0.89(0.82-0.96)	0.002	0.95(0.89-1.01)	0.079	0.76(0.62-0.92)	0.005	0.99(0.92-1.07)	0.788	0.9(0.79-1.02)	0.086
	Quintile4	0.88(0.8-0.95)	0.001	0.91(0.85-0.97)	0.003	0.71(0.58-0.86)	0.001	0.94(0.88-1.02)	0.127	0.8(0.71-0.92)	0.001
	Quintile5	0.83(0.76-0.91)	<0.001	0.97(0.91-1.04)	0.343	0.71(0.57-0.87)	0.001	1.02(0.95-1.11)	0.568	0.89(0.78-1.02)	0.101
**Spermidine**	Quintile1	1.00 (ref.)	-	1.00 (ref.)	-	1.00 (ref.)	-	1.00 (ref.)	-	1.00 (ref.)	-
	Quintile2	0.85(0.79-0.92)	<0.001	0.93(0.88-0.99)	0.029	0.89(0.73-1.08)	0.245	0.96(0.9-1.04)	0.313	0.85(0.75-0.97)	0.013
	Quintile3	0.87(0.8-0.94)	0.001	0.94(0.88-0.99)	0.04	0.82(0.67-1.01)	0.057	0.99(0.92-1.06)	0.716	0.8(0.71-0.91)	0.001
	Quintile4	0.86(0.79-0.93)	<0.001	0.94(0.88-0.99)	0.037	0.83(0.68-1.01)	0.069	0.98(0.91-1.06)	0.615	0.83(0.73-0.94)	0.004
	Quintile5	0.83(0.76-0.91)	<0.001	0.96(0.9-1.03)	0.236	0.82(0.66-1.01)	0.065	1(0.93-1.08)	0.932	0.88(0.77-1.01)	0.064
**Spermine**	Quintile1	1.00 (ref.)	-	1.00 (ref.)	-	1.00 (ref.)	-	1.00 (ref.)	-	1.00 (ref.)	-
	Quintile2	0.96(0.89-1.04)	0.31	0.97(0.91-1.03)	0.262	0.99(0.82-1.21)	0.955	0.99(0.93-1.07)	0.88	0.89(0.79-1.01)	0.074
	Quintile3	0.93(0.85-1)	0.06	0.91(0.86-0.97)	0.004	0.95(0.78-1.17)	0.649	0.94(0.87-1.01)	0.104	0.85(0.75-0.97)	0.013
	Quintile4	0.93(0.86-1.01)	0.09	0.93(0.87-0.99)	0.027	0.88(0.71-1.08)	0.206	0.96(0.89-1.03)	0.275	0.86(0.75-0.97)	0.015

	Quintile5	0.94(0.86-1.02)	0.147	0.97(0.9-1.03)	0.321	0.84(0.67-1.05)	0.118	1.02(0.94-1.1)	0.667	0.86(0.76-0.99)	0.034
**Putrescine**	Quintile1	1.00 (ref.)	-	1.00 (ref.)	-	1.00 (ref.)	-	1.00 (ref.)	-	1.00 (ref.)	-
	Quintile2	0.87(0.81-0.95)	0.001	1.00(0.94-1.07)	0.956	0.88(0.72-1.07)	0.207	1.01(0.94-1.09)	0.717	0.97(0.86-1.1)	0.655
	Quintile3	0.87(0.81-0.95)	0.001	0.91(0.85-0.96)	0.002	0.76(0.62-0.94)	0.009	0.92(0.86-0.99)	0.029	0.86(0.76-0.98)	0.022
	Quintile4	0.9(0.83-0.97)	0.007	0.98(0.92-1.05)	0.597	0.91(0.75-1.11)	0.367	0.98(0.91-1.06)	0.617	0.95(0.84-1.08)	0.439
	Quintile5	0.87(0.8-0.95)	0.002	0.95(0.89-1.02)	0.169	0.81(0.66-1.00)	0.05	0.97(0.89-1.04)	0.369	0.93(0.82-1.07)	0.317

Cox proportional hazards regression adjusted for age, sex, Ethnicity (White European, mixed, Asian, black, others), Townsend deprivation index (least deprived, moderate deprived, most deprived), Education status (College or university degree, others), Systolic Blood Pressure, Body mass index, Physical activity (low and high level), Smoking status (Never, Former, Current), alcohol status (Never, Former, Current), sleep duration(<7h, 7-8h, >8h), energy, Hypertension, Diabetes, Hypercholesteremia, Drug use (Antihypertensive, Lipid treatment, Insulin treatment)

Sensitivity analysis Table S3: Sensitivity analyses adjusting for potential effect mediators (CHD and stroke predisposition score).

		all-cause mortality※		CVD events※		CVD death※		CHD ∫		Stroke＆	
		**HR (95% CI)**	***P value***	**HR (95% CI)**	***P value***	**HR (95% CI)**	***P value***	**HR (95% CI)**	***P value***	**HR (95% CI)**	***P value***
**Dietary polyamines**	Quintile1	1.00 (ref.)	-	1.00 (ref.)	-	1.00 (ref.)	-	1.00 (ref.)	-	1.00 (ref.)	-
	Quintile2	0.8(0.74-0.87)	<0.001	0.89(0.84-0.95)	<0.001	0.68(0.57-0.83)	<0.001	0.94(0.88-1.01)	0.099	0.79(0.7-0.89)	<0.001
	Quintile3	0.87(0.81-0.93)	<0.001	0.9(0.85-0.96)	0.001	0.77(0.64-0.92)	0.005	0.95(0.88-1.01)	0.116	0.84(0.75-0.95)	0.004
	Quintile4	0.84(0.78-0.91)	<0.001	0.87(0.82-0.92)	<0.001	0.71(0.59-0.86)	<0.001	0.9(0.84-0.96)	0.003	0.76(0.67-0.85)	<0.001
	Quintile5	0.82(0.76-0.89)	<0.001	0.92(0.87-0.97)	0.005	0.74(0.62-0.9)	0.002	0.96(0.9-1.03)	0.3	0.84(0.75-0.95)	0.005
**Spermidine**	Quintile1	1.00 (ref.)	-	1.00 (ref.)	-	1.00 (ref.)	-	1.00 (ref.)	-	1.00 (ref.)	-
	Quintile2	0.83(0.77-0.89)	<0.001	0.9(0.85-0.95)	<0.001	0.84(0.7-1.01)	0.064	0.94(0.88-1.00)	0.063	0.81(0.72-0.91)	<0.001
	Quintile3	0.84(0.78-0.91)	<0.001	0.91(0.85-0.96)	0.001	0.79(0.65-0.95)	0.014	0.96(0.9-1.03)	0.281	0.77(0.69-0.87)	<0.001
	Quintile4	0.84(0.78-0.9)	<0.001	0.9(0.85-0.96)	0.001	0.8(0.66-0.97)	0.02	0.96(0.89-1.02)	0.2	0.78(0.69-0.88)	<0.001
	Quintile5	0.84(0.78-0.91)	<0.001	0.94(0.89-1.00)	0.051	0.82(0.68-0.99)	0.036	0.99(0.93-1.07)	0.87	0.85(0.76-0.96)	0.008
**Spermine**	Quintile1	1.00 (ref.)	-	1.00 (ref.)	-	1.00 (ref.)	-	1.00 (ref.)	-	1.00 (ref.)	-
	Quintile2	0.91(0.85-0.98)	0.017	0.94(0.89-1.00)	0.054	0.92(0.77-1.11)	0.387	0.98(0.92-1.05)	0.608	0.87(0.77-0.97)	0.015
	Quintile3	0.88(0.82-0.95)	0.001	0.89(0.84-0.95)	<0.001	0.88(0.73-1.06)	0.185	0.93(0.87-1.00)	0.05	0.82(0.73-0.92)	0.001
	Quintile4	0.89(0.82-0.96)	0.002	0.91(0.85-0.96)	0.001	0.81(0.67-0.99)	0.035	0.94(0.88-1.01)	0.083	0.83(0.74-0.93)	0.002

	Quintile5	0.9(0.84-0.98)	0.01	0.96(0.9-1.02)	0.172	0.77(0.63-0.93)	0.008	1.01(0.95-1.09)	0.707	0.88(0.78-0.99)	0.03
**Putrescine**	Quintile1	1.00 (ref.)	-	1.00 (ref.)	-	1.00 (ref.)	-	1.00 (ref.)	-	1.00 (ref.)	-
	Quintile2	0.85(0.79-0.92)	<0.001	0.94(0.88-0.99)	0.026	0.81(0.68-0.97)	0.025	0.96(0.9-1.03)	0.227	0.88(0.79-0.99)	0.037
	Quintile3	0.86(0.8-0.93)	<0.001	0.85(0.8-0.9)	<0.001	0.71(0.59-0.86)	<0.001	0.88(0.82-0.94)	<0.001	0.8(0.71-0.9)	<0.001
	Quintile4	0.87(0.81-0.94)	<0.001	0.92(0.87-0.98)	0.008	0.84(0.7-1.01)	0.058	0.94(0.88-1.00)	0.058	0.87(0.77-0.98)	0.022
	Quintile5	0.85(0.79-0.92)	<0.001	0.89(0.84-0.95)	<0.001	0.75(0.63-0.9)	0.002	0.91(0.85-0.97)	0.005	0.88(0.78-0.98)	0.024

※：Cox proportional hazards regression adjusted for age, sex, Ethnicity (White European, mixed, Asian, black, others), Townsend deprivation index (least deprived, moderate deprived, most deprived), Education status (College or university degree, others), Systolic Blood Pressure, Body mass index, Physical activity (low and high level), Smoking status (Never, Former, Current), alcohol status (Never, Former, Current), sleep duration(<7h, 7-8h, >8h), energy, Hypertension, Diabetes, Hypercholesteremia, Drug use (Antihypertensive, Lipid treatment, Insulin treatment)

ʃ：Cox proportional hazards regression adjusted for ※ except stroke predisposition score

&：Cox proportional hazards regression adjusted for ※ except CHD predisposition score

Sensitivity analysis Table S4: Excluding participants with any missing values of covariates.

		all-cause mortality		CVD events		CVD death		CHD		Stroke	
		**HR (95% CI)**	***P value***	**HR (95% CI)**	***P value***	**HR (95% CI)**	***P value***	**HR (95% CI)**	***P value***	**HR (95% CI)**	***P value***
**Dietary polyamines**	Quintile1	1.00 (ref.)	-	1.00 (ref.)	-	1.00 (ref.)	-	1.00 (ref.)	-	1.00 (ref.)	-
	Quintile2	0.8(0.73-0.87)	<0.001	0.91(0.86-0.98)	0.008	0.67(0.54-0.83)	<0.001	0.97(0.9-1.051)	0.481	0.79(0.69-0.9)	<0.001
	Quintile3	0.86(0.79-0.94)	<0.001	0.92(0.87-0.99)	0.021	0.71(0.57-0.88)	0.002	0.99(0.92-1.07)	0.85	0.81(0.71-0.92)	0.001
	Quintile4	0.87(0.8-0.94)	0.001	0.89(0.83-0.95)	<0.001	0.71(0.57-0.88)	0.002	0.94(0.869-1.017)	0.125	0.74(0.64-0.84)	<0.001
	Quintile5	0.82(0.75-0.9)	<0.001	0.96(0.9-1.031)	0.286	0.77(0.62-0.95)	0.016	1.02(0.944-1.104)	0.607	0.85(0.75-0.98)	0.02
**Spermidine**	Quintile1	1.00 (ref.)	-	1.00 (ref.)	-	1.00 (ref.)	-	1.00 (ref.)	-	1.00 (ref.)	-
	Quintile2	0.83(0.76-0.9)	<0.001	0.93(0.87-0.99)	0.038	0.86(0.7-1.07)	0.178	0.99(0.92-1.08)	0.957	0.79(0.69-0.9)	<0.001
	Quintile3	0.84(0.77-0.91)	<0.001	0.94(0.88-0.99)	0.048	0.81(0.65-1.01)	0.056	0.99(0.93-1.08)	0.941	0.78(0.68-0.89)	<0.001
	Quintile4	0.83(0.76-0.91)	<0.001	0.94(0.88-1.01)	0.087	0.76(0.61-0.95)	0.014	1.03(0.95-1.11)	0.52	0.77(0.68-0.88)	<0.001
	Quintile5	0.85(0.78-0.93)	<0.001	0.99(0.93-1.06)	0.888	0.87(0.7-1.08)	0.199	1.07(0.99-1.16)	0.103	0.84(0.74-0.97)	0.013
**Spermine**	Quintile1	1.00 (ref.)	-	1.00 (ref.)	-	1.00 (ref.)	-	1.00 (ref.)	-	1.00 (ref.)	-
	Quintile2	0.91(0.84-0.99)	0.028	0.95(0.89-1.02)	0.133	0.89(0.72-1.1)	0.263	1.01(0.93-1.08)	0.918	0.83(0.73-0.94)	0.004
	Quintile3	0.86(0.79-0.94)	<0.001	0.89(0.84-0.95)	0.001	0.8(0.64-0.99)	0.039	0.94(0.87-1.02)	0.113	0.79(0.7-0.91)	<0.001
	Quintile4	0.89(0.82-0.97)	0.006	0.93(0.87-0.99)	0.038	0.76(0.61-0.95)	0.015	0.98(0.91-1.06)	0.649	0.81(0.71-0.92)	0.001

	Quintile5	0.92(0.85-1.01)	0.063	0.97(0.91-1.04)	0.399	0.75(0.6-0.94)	0.012	1.04(0.96-1.12)	0.36	0.85(0.75-0.98)	0.02
**Putrescine**	Quintile1	1.00 (ref.)	-	1.00 (ref.)	-	1.00 (ref.)	-	1.00 (ref.)	-	1.00 (ref.)	-
	Quintile2	0.89(0.81-0.97)	0.006	0.97(0.91-1.03)	0.316	0.85(0.68-1.05)	0.13	0.99(0.93-1.08)	0.988	0.87(0.77-0.99)	0.041
	Quintile3	0.85(0.78-0.93)	<0.001	0.88(0.82-0.94)	<0.001	0.74(0.59-0.92)	0.007	0.92(0.85-0.99)	0.031	0.76(0.66-0.87)	<0.001
	Quintile4	0.91(0.84-0.99)	0.032	0.94(0.88-1.00)	0.062	0.86(0.69-1.06)	0.161	0.96(0.89-1.04)	0.313	0.84(0.74-0.96)	0.011
	Quintile5	0.87(0.8-0.95)	0.002	0.94(0.88-1.00)	0.06	0.8(0.64-0.99)	0.037	0.96(0.89-1.03)	0.269	0.87(0.79-1.02)	0.093

Cox proportional hazards regression adjusted for age, sex, Ethnicity (White European, mixed, Asian, black, others), Townsend deprivation index (least deprived, moderate deprived, most deprived), Education status (College or university degree, others), Systolic Blood Pressure, Body mass index, Physical activity (low and high level), Smoking status (Never, Former, Current), alcohol status (Never, Former, Current), sleep duration(<7h, 7-8h, >8h), energy, Hypertension, Diabetes, Hypercholesteremia, Drug use (Antihypertensive, Lipid treatment, Insulin treatment)
